# TeCVP: A Time-Efficient Control Method for a Hexapod Wheel-Legged Robot Based on Velocity Planning

**DOI:** 10.3390/s23084051

**Published:** 2023-04-17

**Authors:** Junkai Sun, Zezhou Sun, Jianfei Li, Chu Wang, Xin Jing, Qingqing Wei, Bin Liu, Chuliang Yan

**Affiliations:** 1 School of Mechanical and Aerospace Engineering, Jilin University, Changchun 130025, China; sunjk20@mails.jlu.edu.cn (J.S.);; 2 Beijing Institute of Spacecraft System Engineering, Beijing 100094, China; 3 State Key Laboratory of Robotics and System, Harbin Institute of Technology, Harbin 150006, China

**Keywords:** wheel-legged robot, control method, time efficient, velocity planning

## Abstract

Addressing the problem that control methods of wheel-legged robots for future Mars exploration missions are too complex, a time-efficient control method based on velocity planning for a hexapod wheel-legged robot is proposed in this paper, which is named time-efficient control based on velocity planning (TeCVP). When the foot end or wheel at knee comes into contact with the ground, the desired velocity of the foot end or knee is transformed according to the velocity transformation of the rigid body from the desired velocity of the torso which is obtained by the deviation of torso position and posture. Furthermore, the torques of joints can be obtained by impedance control. When suspended, the leg is regarded as a system consisting of a virtual spring and a virtual damper to realize control of legs in the swing phase. In addition, leg sequences of switching motion between wheeled configuration and legged configuration are planned. According to a complexity analysis, velocity planning control has lower time complexity and less times of multiplication and addition compared with virtual model control. In addition, simulations show that velocity planning control can realize stable periodic gait motion, wheel-leg switching motion and wheeled motion and the operation time of velocity planning control is about 33.89% less than that of virtual model control, which promises a great prospect for velocity planning control in future planetary exploration missions.

## 1. Introduction

The ‘Zhurong’ rover [1] and the ‘Perseverance’ rover [2] successfully landed on Mars and carried out patrol exploration in 2021. For more than two decades, the Mars patrol exploration has gradually developed towards deeper levels, wider areas and longer periods. In the future, missions such as sampling return, manned exploration and detection base construction will be gradually carried out [3]. With the continuous advancement of exploration missions, the exploration area will gradually expand from plains to mountains and riverbeds [4]. 

Most existing Mars rovers adopt a wheeled structure with rocker-bogie suspension [5,6,7]; hence, wheels easily sink into soft ground, and the climbing ability is relatively weak. Although China’s ‘Zhurong’ rover is equipped with active suspension, it can execute special motion such as lifting wheels, crossing obstacles and creeping out of sinkage [8]. Its adaptability to complex terrain is relatively limited, which will make it difficult to meet the requirements of more rugged terrain during future Mars exploration missions. In response to such problems, a variety of legged planet exploration robots have been proposed to adapt to complex and rugged terrain [9,10,11,12], but most legged robots have low speed and insufficient stability, which is impractical to apply to Mars exploration in short period of time. In comparison, wheel-legged robots have the high speed of wheeled rovers and the strong terrain adaptability of legged robots [13], and thus they have great potential with respect to future Mars exploration missions. Many wheel-legged robots have been proposed for future planetary exploration, such as Athlete [14,15,16], Robosimian [17], Sherpa [18], Sherpa TT [19] and Mammoth [20]. The above-mentioned robots mainly adopt wheeled motion, and control methods are roughly the same. The torque or velocity of leg joints is obtained according to the foot force and the torso posture to perform as an active suspension, which can be used in complex and undulating terrains to make sure that all wheels are on the ground while keeping the posture of the torso as stable as possible. In addition, Beijing University of Aeronautics and Astronautics has developed a wheel-legged robot NOROS [21,22], which adopts legged motion mainly. On the basis of fundamental gaits, a self-recovery gait after overturn is designed [23] and a torso trajectory planning method is also proposed based on exponential coordinates in SE(3) space using the foot end impedance control [24].

For the above-mentioned robot, virtual model control (VMC) [25] and similar methods are adopted to establish the relation between torso and legs. VMC is a motion control framework that uses virtual components to generate virtual forces when the virtual components interact with a robot system. VMC can guarantee the overall compliant behavior of the robot while also maintaining a high level of accuracy in trajectory execution. As a result, VMC has been applied on many legged robots successfully, such as HyQ [26] and starlETH [27]. In order to control the legs of the robot, virtual forces need to be distributed to each foot that touches the environment. The distribution is executed via a pseudo-inverse distribution matrix, which causes a large amount of calculation. However, due to the requirements of overall weight and radiation resistance technology, the performance of controllers on Mars exploration probes is far lower than that of the on-board computer for ground applications. In addition, on-board communication, navigation, perception and other tasks run in parallel, so control method needs to meet time-efficient requirements. Control methods of the above-mentioned robots require a relatively large amount of calculation, leading to lower operating efficiency, which means they will have certain limitations in future Mars exploration missions. 

In addition, the mobile robot trajectory tracking control problem and the gait planning and control problem are well studied, such as the velocity vector control method [28] and the limited integrator anti-windup approach [29]. However, both problems need to be solved with one control method for wheel-legged robots to simplify the control framework. What is more, legged systems should realize soft interactions with an environment to ensure robustness against disturbances and protect hardware from damage through unexpected collisions. To address this issue, impedance control [30] is used to realize stable interactions with the environment in many studies. Over the years, impedance control has attracted great interest and some new control methods based on impedance control have been developed, such as nested impedance control [31]. Nested impedance control can be realized by combining impedance control and other control loops to gain better control performance and fulfill demands of different robots.

Aiming at the above-mentioned problems, a time-efficient control method based on velocity planning is designed, which is named time-efficient control based on velocity planning (TeCVP). The main contributions of this paper are as follows:

1. This paper reduces computation costs of the hexapod wheel-legged robot control method by using the desired velocity planning strategy instead of force distribution.

2. This paper addresses the problem of different motion modes of the hexapod wheel-legged robot using one control method, which can simplify the framework of the control system.

3. This paper uses contact forces between passive wheels and the ground as a feedforward to maintain sufficient active wheel torque during wheeled motion.

The rest of this paper is organized as follows. In Section 2, a hexapod wheel-legged robot is introduced, which is the main application objective of TeCVP. In Section 3, TeCVP is introduced from three aspects: legged motion, wheeled motion and wheel-legged switching motion. A complexity analysis is carried out in Section 4. In Section 5, simulations are presented. Section 6 is the conclusion.

## 2. Description of the Wheel-Legged Robot

**Figure 1:**
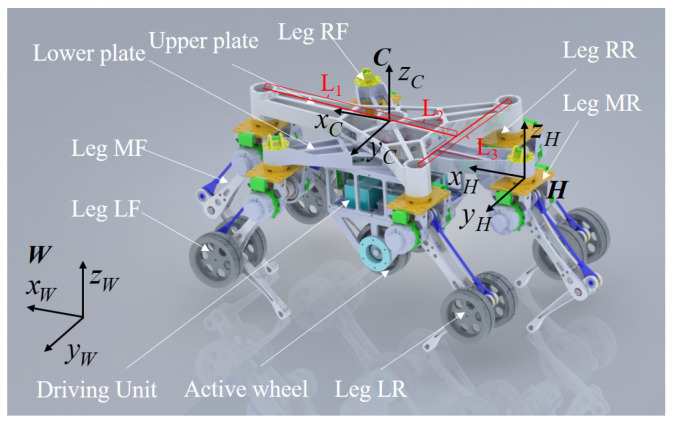
Key coordinate system of the wheel-legged robot.

The TeCVP is designed for an improved model of an existing wheel-legged robot [32] equipped with five wheels and six legs. The main components and key coordinate systems of the wheel-legged robot are shown in Figure 1. The torso is composed of two triangular plates that are the same. The legs are located at the vertices of two plates and named according to the position of the legs, including left front leg (LF), middle front leg (MF), right front leg (RF), left rear leg (LR), middle rear leg (MR) and right rear leg (RR). The distance between the center of a plate and the front vertex L_1_ is 460 mm. The distance between the center of a plate and the line of two rear vertices L_2_ is 250 mm. The distance between the two rear vertices L_3_ is 490 mm. Each leg has two degrees-of-freedom (DOFs). The drive unit of each leg is located at the hip, while the knee joint is driven by a parallelogram structure.

The origin of the torso coordinate system *C* is at the intersection point of the central axis of the plates and the upper surface of the torso. The axis *z^c^* is vertically upward along the central axis of the plates, and the axis *x^c^* is horizontally forward along the longitudinal direction of the torso. On that basis, the axis *y^c^* is determined via the right-hand-side rule. Moreover, the initial states of each axis of the world coordinate system *W* and the leg coordinate system *H* are the same as that of the torso coordinate system *C*. The origin of *H* is at the intersection point of the central section of the hip and the axis of the hip pitch joint.

**Figure 2:**
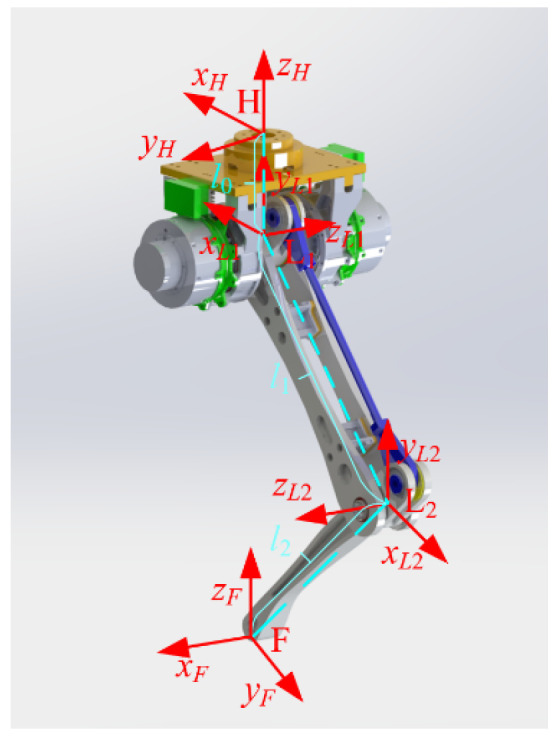
Structure of single leg.

Moreover, the structure of a single leg is shown in Figure 2. The leg coordinate system *H* is at the top of the single leg while the coordinate system *L*_1_ is at the hip of the single leg. The axis *z_L_*_1_ is along the axis of the first joint of the single leg, and the axis *y_L_*_1_ is vertically upward. On that basis, the axis *x_L_*_1_ is determined via the right-hand-side rule. The coordinate system *L*_2_ is at the knee of the single leg and the axis *z_L_*_2_ is along the axis of the second joint of the single leg. The axis *y_L_*_2_ and the axis *x_L_*_2_ are shown in Figure 2. The coordinate system *F* is at the foot end of the single leg. The axis *z_F_* is vertically upward along the normal of the ground. The axis *x_F_* and the axis *y_F_* are shown in Figure 2. Distances between the coordinate system *H* and the coordinate system *L*_1_, the coordinate system *L*_1_ and the coordinate system *L*_2_, the coordinate system *L*_2_ and the coordinate system *F* are *l*_0_ = 120 mm, *l*_1_ = 310 mm and *l*_2_ = 290 mm, respectively, which is shown in Figure 2.

One passive wheel is installed on each knee of legs LF, RF, LR and RR. The active wheel and its driving unit are installed under the torso. The structure’s simplicity and its advantages with respect to electrical power saving are the most important advantages of the passive wheel that is considered in the design of the wheel-legged robot. The simple structure can improve the reliability of the robot and the power saving can prolong the endurance; these factors play an important role on the extension of the exploration area. Hence, four passive wheels are adopted. Because there is only one active wheel, the hexapod wheel-legged robot introduced has a relatively simple structure and high reliability compared to robots with multiple active wheels. In addition, since passive wheels are all installed on knees of legs rather than foot ends and active wheels are suspended during legged motion, unnecessary wear of wheels can be avoided in legged motion, which can prolong the life-span of the robot.

**Figure 3:**
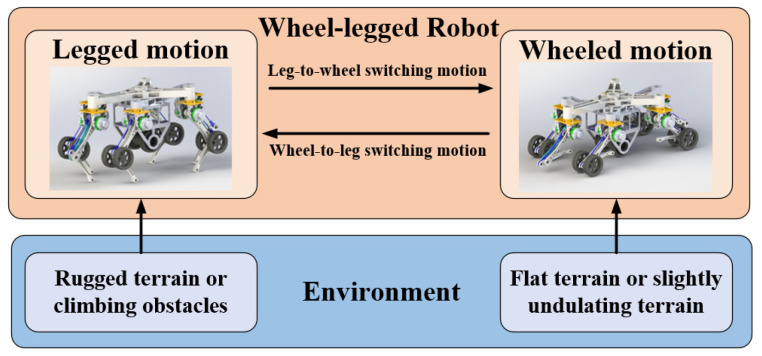
Relationship between motion mode and terrain type.

The robot has four motion modes, wheeled motion, legged motion, leg-to-wheel switching motion and wheel-to-leg switching motion, as shown in Figure 3. When the robot needs to cross rugged terrains or climb obstacles, legged motion mode is adopted. In legged motion mode, all wheels are suspended and the torso is moved by legs. If a foot touches the ground, the leg is in the stance phase. Otherwise, the leg is in the swing phase. When the terrain is flat or slightly undulating, wheeled motion is adopted. Active wheels mainly support the torso and provide driving torque, while passive wheels maintain the posture of the torso. During the leg-to-wheel switching motion, the height of the torso is decreased to make the active wheels come into contact with the ground and the six feet are lifted to make the passive wheels come into contact with the ground sequentially. In contrast, the torso is lifted and the six feet touch the ground again during the wheel-to-leg switching motion mode. In summary, the state of the legs can be divided into three states: two joint support state (during stance phase of legged motion), one joint support state (during wheeled motion) and suspending state (during swing phase of legged motion). The state of the legs in the leg-to-wheel switching motion and the wheel-to-leg switching motion includes those three states too.

## 3. Control Method Based on Velocity Planning

### 3.1. Legged Motion

#### 3.1.1. Stance Phase

During the stance phase, the torso is moved by the legs according to the desired trajectory, while ensuring that the torso’s posture is relatively stable. Since a single leg has two active DOFs, the leg cannot move along the axis *y^H^* of leg coordinate system *H*. Therefore, the torso cannot move along the axis *y^W^* of world coordinate system *W* or rotate around the axis *z^W^* of world coordinate system *W* via the rotation of the leg joint. 

To further discuss the velocity of the robot, consider that the position of the foot with respect to the world origin reads as

(1) PwfW=PwcW+PchW+PhfW

where PwcW is the position of the center of mass (CoM) with respect to the world origin, PchW is the position of the hip of the leg with respect to the CoM, PhfW is the position of the foot with respect to the leg origin. The left superscript consisting of the coordinate system symbol in parentheses indicates a variable expressed in a specific coordinate system frame. Hence,

(2) PwfW=PwcW+RCWPchC+RCWRHCPhfH

where $R_{C}^{W}$ is the transformation matrix from torso coordinate system *C* to world coordinate system *W*, PchC is the position of the hip of the leg with respect to the CoM in torso coordinate system *C*, $R_{H}^{C}$ is the transformation matrix from leg coordinate system *H* to torso coordinate system *C* and PhfH is the position of the foot with respect to the hip of the leg in leg coordinate system *H*.

The derivative of foot position then reads

(3) P˙wfW=P˙wcW+R˙CWPchC+RCWP˙chC+R˙CWRHCPhfH+RCWR˙HCPhfH+RCWRHCP˙hfH

where P˙wcW is the derivative of CoM position, P˙chW is the derivation of hip position and P˙hfW is the derivative of foot position with respect to the leg origin. Since the robot is regarded as a rigid body, P˙chC=0 and ${\dot{R}}_{H}^{C}=0$. Then, Equation (3) can be rewritten as

(4) P˙wfW=P˙wcW+R˙CWPcfC+RHWP˙hfH

Assume that the actual position and posture of the torso in world coordinate system W are

(5) PW=xW,zW,αW,βWT

where xW,zW are the position of CoM, and αW,βW are the roll angle and pitch angle of the torso, respectively. The deviation of the position and posture of the torso is shown in Equation (6).

(6) ΔPW=PdW−PW=xdW−xW,zdW−zW,αdW−αW,βdW−βWT

where PdW=xWd,zWd,αWd,βWd is the desired position and posture of the torso. The desired velocity and angular velocity of the torso in world coordinate system W are shown in Equation (7).

(7) VW=vW,wW=K∗•ΔPW

where vW∈R1×2,wW∈R1×2 are the desired velocity and angular velocity of the torso respectively, $K\in R^{1\times 4}$ is the coefficient vector of the sliding mode surface and the symbol ∗• represents the position multiplication of the vector, such as $x=\left[x_{1},x_{2}\right]$, $y=\left[y_{1},y_{2}\right]$, then x∗•y=x1y1,x2y2.

As the foot is stationary on the ground during the stance phase, the velocity of the foot remains zero in world coordinate system *W*, i.e., P˙Wwf=0 in (4). Hence, the linear velocity of each foot end can be obtained as shown in Equation (8).

(8) VHfd_i=P˙Hhf=−RHWvW+wW×PWcf

where VHfd_i∈R1×2 is the linear velocity of the *i*th foot end. According to the Jacobian matrix of velocity and angular velocity of each joint, the actual velocity of foot ends in the torso coordinate system can be calculated, and the velocity deviation of foot ends is shown in Equation (9).

(9) ΔVH=VHf_i−VHfd_i

where VHf_i∈R1×2 is the actual velocity of the *i*th foot end. According to impedance control, the desired force of the *i*th foot end is shown in Equation (10).

(10) FHf=Kd∗•ΔVH+Kk∗•∫ΔVH

where $K_{d}\in R^{1\times 2},K_{k}\in R^{1\times 2}$ are the damping coefficient vector and the stiffness coefficient vector of impedance control, respectively. According to the Jacobian matrix of force, the torque of each joint can be calculated as shown in Equation (11).

(11) τ=JHFFHfT

where $\tau \in R^{2\times 1}$ is the torque vector, and JHF∈R2×2 is the Jacobian matrix of force. 

**Figure 4:**
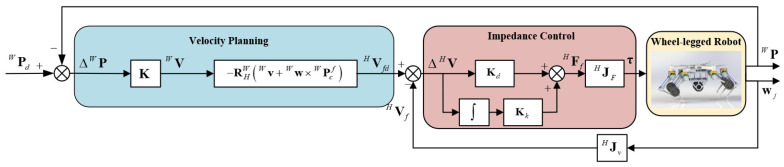
Control block diagram of the stance phase. The control block diagram has two main sub-blocks: sub-block of velocity planning and sub-block of impedance control. The sub-block of velocity planning plans the desired velocity of the torso and the desired velocity of the foot. The sub-block of impedance control calculates the torque of joints to control the wheel-legged robot to execute the desired motion.

In accordance with Equations (5)–(11), the control block diagram of the stance phase during legged motion is shown in Figure 4. In the sub-block of velocity planning, the desired velocity of torso VW is planned with the deviation of the position and posture of torso ΔPW and the sliding surface coefficient K, as shown in Equations (5)–(7). After that, the desired velocity of foot VHfd is planned according to Equation (8). In the sub-block of impedance control, the deviation of foot velocity ΔVH and its integration are used to obtain the desired force of foot FHf according to Equations (9) and (10). Finally, the torque of the joints can be calculated with the Jacobian matrix of force JHF and the desired force of foot FHf according to Equation (11). It is noted that the actual velocity of the foot is calculated via the angular velocity of joint $w_{j}$ and the Jacobian matrix of velocity JHv which is the transposition of the Jacobian matrix of force JHF.

#### 3.1.2. Swing Phase

During the swing phase, foot ends are lifted off the ground and moved along a specific trajectory to the next footing point smoothly. The actual position of the foot end can be calculated according to angles of the joints and the forward kinematic equations. The deviation from the desired trajectory is

(12) ΔPHf=PHdf−PHf

where PHdf∈R1×2 is the actual position of the foot end in the swing phase, PHf∈R1×2 is the desired position of the foot end. Then the desired force of the foot end should be

(13) FHff=Kkf∗•ΔPHf+Kdf∗•ddtΔPHf

where $K_{k}^{f}\in R^{1\times 2}$ is the stiffness coefficient vector, $K_{d}^{f}\in R^{1\times 2}$ is the damping coefficient vector. In accordance with the Jacobian matrix of force, the torque of each joint can be calculated.

(14) τ=JHFfFHffT

where JHFf is the Jacobian matrix of force. 

**Figure 5:**
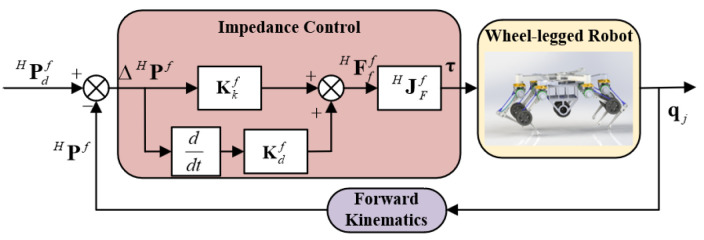
Control block diagram of the swing phase. The sub-block of impedance control calculates the torque of joints to make the swing leg move smoothly and stably.

In accordance with Equations (12)–(14), the control diagram of the swing phase is shown in Figure 5. The deviation of foot position ΔPHf is calculated via the desired position of foot PHdf and the actual position of foot PHf according to Equation (12). The actual position of the foot can be obtained with the joint position $q_{j}$ and the forward kinematics of the single leg, which is not introduced in detail in this paper. In the sub-block of impedance control, the desired force of foot FHff is calculated via the deviation of foot position ΔPHf and its differential according to Equation (13). Finally, the torque of joint τ is obtained via the Jacobian matrix of force JHFf according to Equation (14).

### 3.2. Wheeled Motion

When the robot is in the wheeled motion mode, the torso is supported by the passive wheels and active wheel. The state of the legs can be regarded as a single-joint stance phase, and the control method is similar to that of the stance phase. Since legs only use the pitch joint at the hip to support the robot during wheeled motion, the DOFs of the torso are less than that in legged motion mode. Assume that the position and posture of the torso during wheeled motion are

(15) PWs=zWs,βWsT

where zWs is the position of the torso’s CoM in the world coordinate system; βWs is the pitch angle of the torso. The deviation of the position and pitch angle of the torso is shown in Equation (16).

(16) ΔPWs=PWds−PWs=zWds−zWs,βWds−βWsT

where PWds=zWds,βWds is the desired position and pitch angle of the torso. The desired velocity and angular velocity of the torso in the world coordinate system are shown in Equation (17).

(17) VWs=vWs,wWsT=Ks∗•ΔPWs

where vWs,wWs is the desired velocity and angular velocity of the torso’s CoM, $K^{s}\in R^{1\times 2}$ is the coefficient vector of sliding mode surface. 

Similar to the stance phase, the desired velocity of the knee reads

(18) VHfd_is=−RHWvWs+wWs×PWf_is

where VHfd_is is the linear velocity of the ith knee; PHf_is is the coordinate of the *i*th knee in the torso coordinate system. With the Jacobian matrix of velocity and the joint’s angular velocity, the actual velocity of the knees in the torso coordinate system can be calculated. For one leg, the knee velocity deviation is shown in Equation (19).

(19) ΔVHs=VHf_is−VHfd_is

where $V_{f\_i}^{s}$ is the actual velocity of the *i*th knee. With the impedance control, the force of the *i*th knee is shown in Equation (20).

(20) FHfs=Kds⋅ΔVHs+Kks⋅∫ΔVHs

where $K_{d}^{s},K_{k}^{s}$ are the damping coefficient and the stiffness coefficient. With the angle of the hip joint, the torque can be calculated in wheeled motion. 

However, since only the active wheel provides driving force during wheeled motion, once the terrain undulates the active wheel is easily suspended so that the driving force decreases. Therefore, during wheeled motion, the desired contact forces between the passive wheels and the ground are used to calculate a feedforward of the torque of the hip joint to ensure that the active wheel can touch the ground at any moment. The feedforward item is calculated as shown in Equation (21).

(21) $$\tau_{f}=F_{c}\cos\alpha$$

where $F_{c}$ is the contact force between the passive wheels and the ground and α is the joint position of the hip joint. With the Jacobian matrix of force, the torque of each joint can be calculated as shown in Equation (22).

(22) τ=JHFsFHfsT+τf

where JHFs∈R2×2 is the Jacobian matrix of force. 

**Figure 6:**
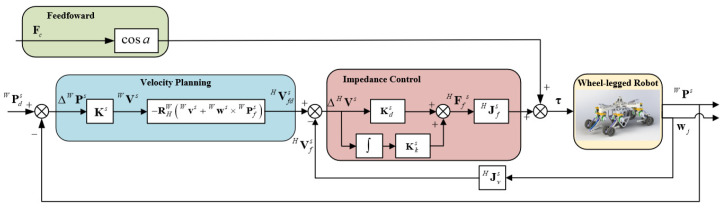
Control block diagram of the wheeled motion. The control block diagram has three main sub-blocks: sub-block of feedforward, sub-block of velocity planning, sub-block of impedance control. The main purpose of sub-block of feedforward is to maintain the contact between the wheels and the ground. The sub-block of velocity planning and the sub-block of impedance control are similar to those of the control block diagram of the stance phase.

In accordance with Equations (15)–(22), the control block diagram of the wheeled motion is shown in Figure 6. The sub-block of velocity and the sub-block of impedance control are similar to those of Figure 4. In the sub-block of feedforward, a feedforward item obtained by the contact force $F_{c}$ and the joint angle of hip joint α adds to the torque of the hip joint in accordance with Equation (22).

### 3.3. Wheel-Leg Switching Gait Planning

The position of the torso’s CoM is relatively high during legged motion, and all wheels are suspended. During the leg-to-wheel switching process, the CoM of the torso needs to be lowered to make the active wheel fully land on the ground and provide sufficient driving force. In addition, the foot ends need to be lifted to a safe position when passive wheels touch the ground. On the other hand, the torso needs to be raised in the wheel-to-leg switching process, and the transition from wheel support to leg support is completed. The planning of the leg sequence is presented in the following section. Taking the smoothness and flexibility requirements of the switching process into account, the trajectory of CoM and that of the foot end adopt a quintic curve. The expression of the desired trajectory will not be introduced in detail.

**Figure 7:**
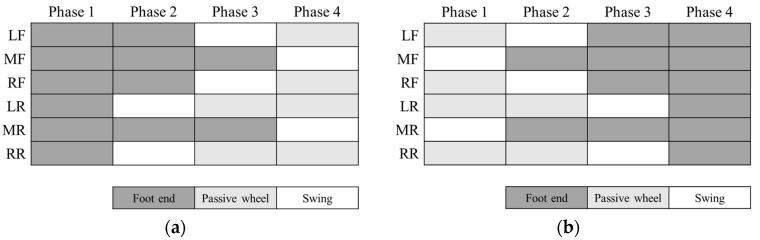
Leg sequence of switching process. (**a**) leg-to-wheel; (**b**) wheel-to-leg.

At the beginning of the leg-to-wheel switching process, the hexapod wheel-legged robot is in six-leg support. In order to reduce the change in the roll angle of the torso, the leg-to-wheel switching process should try to ensure legs that are axisymmetric about the axis *x^C^* of torso coordinate system *C* are in the same state. Figure 7a shows the leg sequence of the leg-to-wheel switching process. In Phase 1 of Figure 7a, all legs touch the ground. In Phase 2 of Figure 7a, legs LR and RR are raised and switched to the wheel support state, while the remaining four legs support the torso in its descent so that the active wheel touches the ground. In Phase 3 of Figure 7a, legs LF and RF are lifted up and make passive wheels touch the ground. Finally, legs MR and MF are lifted up and all passive wheels touch ground in Phase 4 of Figure 7a. 

Figure 7b shows the leg sequence of the wheel-to-leg switching process. In Phase 1 of Figure 7b, four passive wheels support the robot and legs MF and MR are suspended. Since there is no transition process from wheel support to leg support for legs at both ends of the torso, MF and MR can be converted to the leg support state first in Phase 2 of Figure 7b, so that they can provide a certain amount of support force and reduce the load of intermediate legs. After that, two intermediate legs LF and RF are selected to execute the conversion in Phase 3 of Figure 7b, and finally the switching of the two remaining legs LR and RR is completed in Phase 4 of Figure 7b. After all legs complete the switching from wheel support to leg support, the position of the CoM and the posture of the torso are adjusted to the initial state of legged motion. 

## 4. Complexity Analysis

### 4.1. Time Complexity

Calculations of VPC are composed of addition and subtraction of vector, bit multiplication, cross multiplication and matrix multiplication. The time complexity depends on the calculation for joint torque in accordance with the Jacobian matrix based on the desired forces of feet, during which a two-dimensional matrix is multiplied by a two-dimensional column vector. The time complexity of TeCVP is *O*_1_(*n*^3^), where *n* = 2. 

For VMC, virtual spring and virtual damp are set at the center of the body and the expected force and moment at the CoM of the torso are calculated in accordance with the position and posture deviation of the torso as shown in Equation (23).

(23) $$F_{vmc}=K_{vmc}^{k}*\Delta P_{c}+K_{vmc}^{d}*\frac{d\Delta P_{c}}{dt}$$

where $F_{vmc}$ is the expected force and moment of the torso, $K_{vmc}^{k}$ is the stiffness coefficient of the virtual spring, $\Delta P_{c}$ is the position and posture deviation of the torso and $K_{vmc}^{d}$ is the damping coefficient of the virtual damp. With the relationship between the foot force and the force and moment of the torso, it can be obtained that

(24) $$F=T*F_{foot}$$

where T is the transform matrix from the foot force to the torso force and $F_{foot}$ is the foot force.

Then, the foot force can be obtained via the inverse of T and the expected force and moment of torso $F_{vmc}$, as shown in Equation (25).

(25) $$F_{foot}=T^{\prime }*F_{vmc}$$

where $T^{\prime }$ is the inverse of T and is called the force distribution matrix. Finally, torques of joints can be calculated based on foot force via the Jacobian matrix. The force distribution matrix is obtained via the LU decomposition method.

Taking the stance phase of tripod gait as an example, forces of three legs need to be allocated, and the coefficient matrix is a six-dimensional matrix. Therefore, the time complexity of VMC is *O*_2_(*n*^3^), where *n* = 6. Because the swing phase control method of TeCVP is similar to that of the virtual model control, the time complexity of two methods is approximately the same. Therefore, TeCVP is far less time-complex than VMC and can save computing time during long-term operation.

### 4.2. Times of Basic Operations

Analyzing the instances of multiplication and addition of control methods can intuitively reflect the calculation amount and enable one to evaluate the efficiency of the control methods. As the control methods of swing phase of TeCVP and VMC are the same, the instances of multiplication and addition of TeCVP and VMC in the stance phase are analyzed. Regarding the above introduction to equations of TeCVP, the instances of multiplication and addition in each equation of stance phase are shown in Table 1.

Since the control method in the stance phase of TeCVP is only for single leg control, while three legs touch the ground in the stance phase, the total number of instances of multiplication for the stance phase control method is 42, and that of addition is 39.

During LU decomposition of a matrix with size *n*, the instances of multiplication *N_m_* and addition *N_a_* are shown in Equation (26).

(26) $$\left\{\begin{array}{c} N_{m}=n\left(n-1\right)+\left(n-1\right)\left(n-2\right)+\cdots +2\times 1\\ N_{a}=n\left(n-1\right)+\left(n-1\right)\left(n-2\right)+\cdots +2\times 1 \end{array}\right.$$

where *n* = 6, *N_m_* = 70 and *N_a_* = 70. During VMC, the instances of multiplication and those of addition are much larger than those of LU decomposition. Therefore, the number of instances of basic operations of TeVPC is relatively small compared to that of VMC. Table 2 shows the instances of basic operations during main process of VMC.

## 5. Simulations

**Figure 8:**
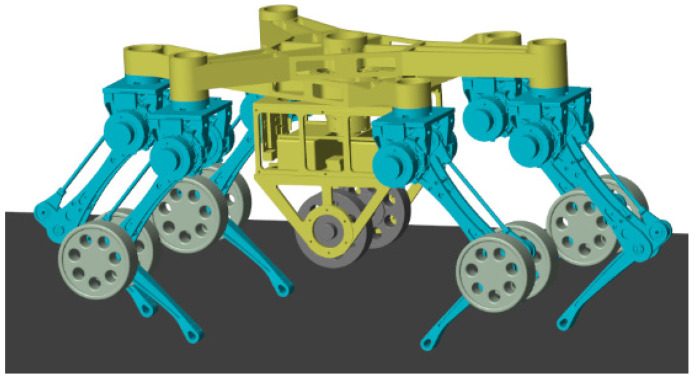
Simulation model of the wheel-legged robot.

In order to verify the effectiveness of TeCVP and the complexity analysis, a simulation model is established using Matlab/Simulink, as shown in Figure 8. On that basis, simulations and comparisons of tripod gait motion, wheel-leg switching motion using TeCVP and VMC were conducted to prove the feasibility of TeCVP. Because VMC is mainly used for legged motion, the simulation of wheeled motion was only conducted using TeCVP, which can also prove the feasibility of TeCVP in wheeled motion. 

The coefficients of TeCVP and VMC controller are tuned according to the results of simulations. Considering that the torso should move along the desired trajectory smoothly in the stance phase of legged motion and wheeled motion, the coefficient vector of sliding mode surface and stiffness vector of impedance control are set to an original value according to the mass of the robot. After that, the above-mentioned two coefficients are optimized in accordance with the performance in the simulation. The damping vector of impedance control is then set to avoid the vibration. In addition, the stiffness vector of impedance control in the swing phase is set in accordance with the mass of the leg and optimized in accordance with the tracking accuracy of foot trajectory. The damping vector of impedance control in the swing phase is tuned to avoid leg vibration. For VMC, only two coefficient vectors need to be tuned. The virtual stiffness vector is tuned to support the whole wheel-legged robot and gain a better tracking accuracy of torso trajectory, while the virtual damping vector is tuned to avoid the vibration in accordance with the performance in simulation. The value of each coefficient is shown in Table 3 and Table 4. The values of [1000;1000;1000;1000] of K in Table 3 mean those of four elements of the vector, and the values of each element of the other coefficient vectors are also indicated in Table 3 and Table 4, respectively.

### 5.1. Tripod Gait Motion

**Figure 9:**
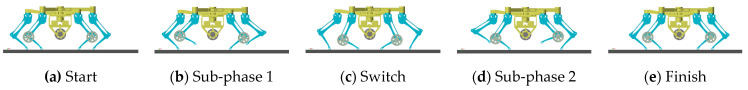
Simulation of tripod gait motion using TeCVP.

During tripod gait motion, three legs installed on the same plate move synchronously. The two sets of leg alternately cycle in the swing phase and the stance phase. Legs move forward along the desired trajectory in the swing phase, and legs in the stance phase support the torso and push the torso forward. These two phases cooperate to realize the forward movement of the robot. The simulation of tripod gait motion is shown in Figure 9. 

**Figure 10:**
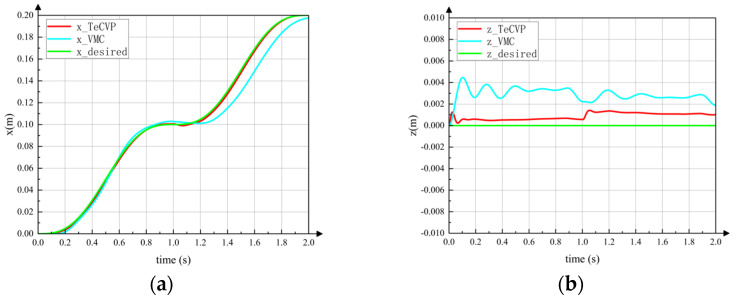
Displacement of torso in tripod gait. The same desired trajectory as the green line in the figures is tracked using the TeCVP and VMC. The blue line is the actual trajectory using VMC, while the red line is the actual trajectory using VMC. (**a**) Displacement x. (**b**) Displacement z.

Figure 10 shows the horizontal displacement of the torso’s CoM during tripod gait motion using TeCVP and VMC, where 1 s is the switching time, as shown in (c) of Figure 9, and 2 s is the end time. The red line is the trajectory using TeCVP. The blue line is the trajectory using VMC. The green line is the desired trajectory. It can be seen from curves in Figure 10a that the CoM of the torso moves smoothly along the horizontal direction using TeCVP, and the horizontal velocities of the CoM at the start, switching and end moments are relatively small, which can ensure that the robot completes the state transition stably. In addition, the error of horizontal displacement of the torso’s CoM using TeCVP is much smaller than that using VMC. Hence, TeCVP can improve the accuracy of trajectory tracking during tripod gait relatively. The vertical displacements of the torso’s CoM during tripod gait using TeCVP and VMC are shown in Figure 10b. TeCVP can also decrease the fluctuation of the torso’s CoM and maintain stability during tripod gait.

In order to verify the complexity analysis in Section 4, the running time of TeCVP is recorded and compared with that of VMC. Thirty simulations are carried out for TeCVP and VMC on a computer with Intel Core i7-10510U @1.8 GHz. Fifteen simulations are conducted under same conditions for one period of tripod gait which takes 2 s and the frequency of the control algorithm is 0.001 s. Through comparison, it can be found that the mean running time of VMC is 0.2967 s in one period of simulation of tripod gait, while TeCVP only needs 0.2216 s. The running time is reduced by about 33.89% and the calculation consumption of the control method is reduced significantly. In addition, the variances of the two sets are 1.0 × 10^−6^ and 1.6 × 10^−4^, which means the running time of the simulations is relatively stable and can reflect the performance of the control method accurately.

### 5.2. Switching Motion

**Figure 11:**
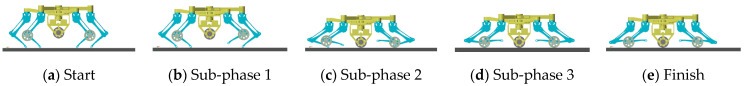
Simulation of leg-to-wheel switching motion using TeCVP.

**Figure 12:**
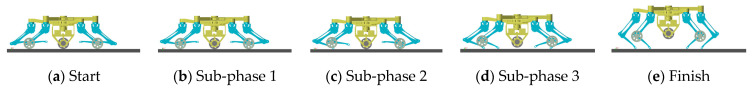
Simulation of wheel-to-leg switching motion using TeCVP.

According to Section 3, the process of leg-to-wheel switching is shown in Figure 11. In the simulation, the robot is in hexapod support at the beginning (phase (a)), and all wheels are suspended. During phase (b), the two rear legs LR,RR located at the right side of torso in the simulation are raised, and the four remaining legs make the torso descend until the active wheel touches the ground. Meanwhile, the angles of the hip joints and knee joints of legs LR and RR are adjusted until the passive wheels touch the ground and foot ends are retracted to a safe position. In phase (c), the two front legs LF and RF on the left side of the torso are raised and conduct the same motion as the legs LR and RR in phase (b). During phase (d), the legs at both ends are raised, and the foot ends are retracted to a safe position at the same time in phase (e). According to the leg sequence planning in Section 3, the simulation of the wheel-to-leg switching process is shown in Figure 12. In the simulation, legs at both ends are first converted to the leg support state. Then, legs LF and RF and legs LR and RR are converted to leg support state successively. Finally, the torso is raised to the initial height during legged motion. The entire wheel-to-leg switching process can be regarded as the inverse process of the leg-to-wheel switching process. Through simulation, it can be found that the wheel-to-leg motion presented in Section 3 can effectively complete the conversion from wheeled configuration to legged configuration stably.

**Figure 13:**
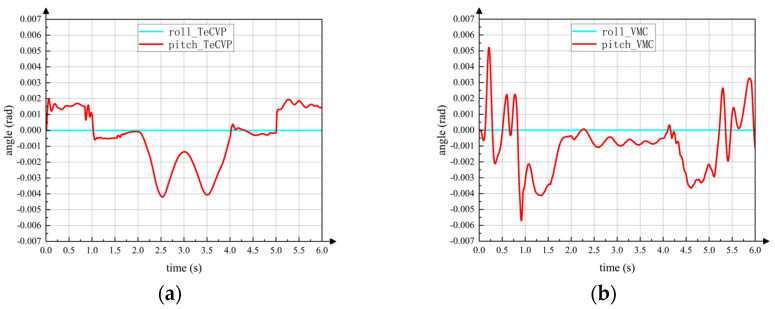
Posture of torso during switching motion (**a**) TeCVP; (**b**) VMC.

In addition, from the screenshots of simulation it can be seen that the posture change of the torso is relatively small during the switching process. Figure 13a,b show the posture of the torso during the switching process using TeCVP and VMC, respectively. In 0–3 s, the wheel-legged robot executes the transformation from the legged configuration into the wheeled configuration, while the transformation from the wheeled configuration into the legged configuration is executed in 3–6 s. At 0 s and 3 s, the states of the robot are as shown in Figure 11a,e. In 0–1 s, 1–2 s, and 2–3 s, the states of robot are as shown in Figure 11b–d, respectively. Similarly, at 6 s the state of the robot is as shown in Figure 12e. In 3–4 s, 4–5 s and 5–6 s, the states of the robot are as shown in Figure 12b–d, respectively. Using TeCVP, the pitch angle of the torso is relatively small and stable, as shown in Figure 13a. However, the pitch angle of the torso using VMC is much bigger than that using TeCVP. Hence, TeCVP can complete a more stable transformation between the wheeled configuration and the legged configuration. 

As legs which are axisymmetric about the axis *x^C^* of torso coordinate system *C* are in the same state, the roll angle of the torso remains zero approximately using both control methods. Although the pitch angle changes, the magnitude is smaller than 0.005 rad in simulation. Hence, the motion planning of the leg-to-wheel switching process and the wheel-to-leg switching process in Section 3 can realize smooth conversion from the legged configuration to wheeled configuration.

### 5.3. Wheeled Motion

**Figure 14:**
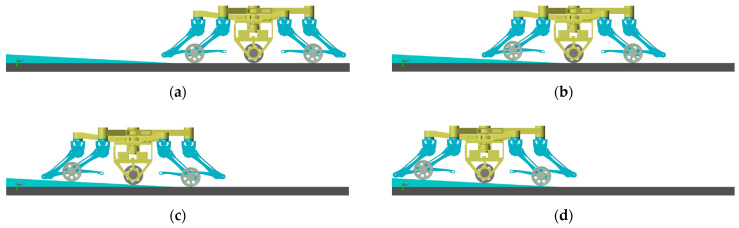
Simulation of wheeled motion (all wheels on slope). (**a**) no wheel on slope; (**b**) front wheels on slope; (**c**) front wheels and active wheel on slope; (**d**) all wheels on slope.

When the robot is in wheeled motion, it is necessary to keep the active wheel in contact with the ground to provide sufficient driving torque. Therefore, a simulation and an analysis of the transition from a flat terrain to a gentle slope in wheeled motion are conducted, as shown in Figure 14. In Figure 14a, the robot runs completely on flat ground, while Figure 14b–d complete the transition from flat terrain to slope for front wheels, active wheel and rear wheels. It can be seen that the active wheel is always in contact with the ground. In addition, the posture of the torso is kept relatively stable. Hence, the robot can adapt to the undulation of the ground.

**Figure 15:**
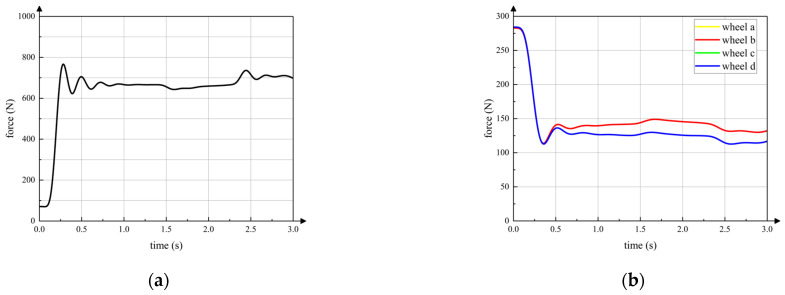
Vertical contact force between wheels and ground (all wheels on slope). (**a**) active wheel; (**b**) passive wheels.

The curve in Figure 15a is the vertical component of the contact force between the active wheel and ground. The horizontal axis is the simulation time. The front wheels, the active wheel and the rear wheels start moving on the slope at 1.4 s, 2.3 s and 2.8 s, respectively, which is same in the following simulations. It can be seen that contact force between the active wheel and the ground remains relatively stable during the entire wheeled motion. The contact force feedforward and posture feedback adjustment strategies can ensure that the active wheel touches ground when the robot passes over the gentle slope. Curves in Figure 15b are vertical contact forces between passive wheels and the ground. Because the front passive wheel a and passive wheel b are symmetrical with respect to the axis *x^C^* of torso coordinate system *C*, the contact forces of passive wheel a and passive wheel b are almost same. Thus, contact force curves of passive wheel a and passive wheel b seem like one curve. In addition, the rear passive wheel c and passive wheel d have same characteristics of passive wheel a and passive wheel b. The contact forces of passive wheels are always within a certain range to keep the active wheel touching the ground.

The pitch angle of the torso changes little during wheeled motion, which means that the posture of the robot can be kept relatively stable on the gentle slope. It can be seen that the torso adjustment strategy during wheeled motion proposed in this paper can ensure that the active wheel is in full contact with the ground on the basis of ensuring stable posture of torso, which can provide sufficient driving force under a gentle slope.

**Figure 16:**
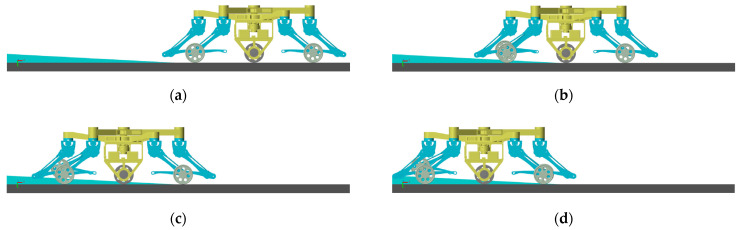
Simulation of wheeled motion (unilateral passive wheels on slope). (**a**) no wheel on slope; (**b**) front wheel on slope; (**c**) front wheel on slope and active wheel on flat ground; (**d**) two wheels on slope.

**Figure 17:**
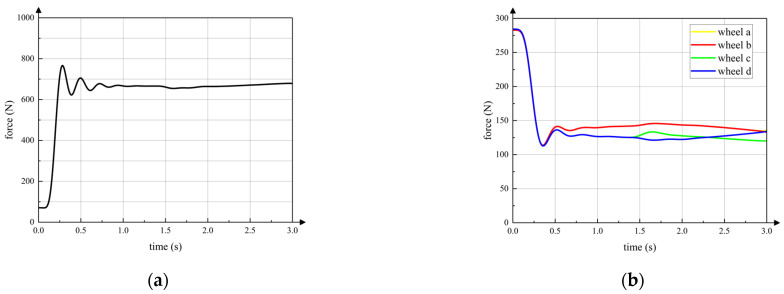
Vertical contact force between wheels and ground (unilateral passive wheels on slope). (**a**) active wheel; (**b**) passive wheels.

In addition, as terrain differs, the passive wheels and active wheel are in different states during wheeled motion. As shown in Figure 16, only unilateral passive wheels move on the slope while the active wheel and other passive wheels are still on the flat terrain. The vertical contact forces between wheels and the ground are shown in Figure 17. Through Figure 17, it can be seen that sufficient driving force can be provided and the torso remains stable.

**Figure 18:**
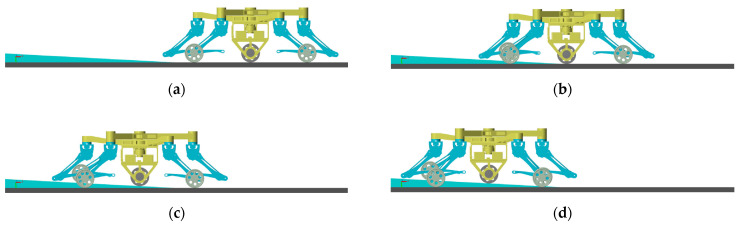
Simulation of wheeled motion (unilateral passive wheels and active wheel on slope). (**a**) no wheel on slope; (**b**) front wheel on slope; (**c**) front wheel and active wheel on slope; (**d**) Three wheels on slope.

**Figure 19:**
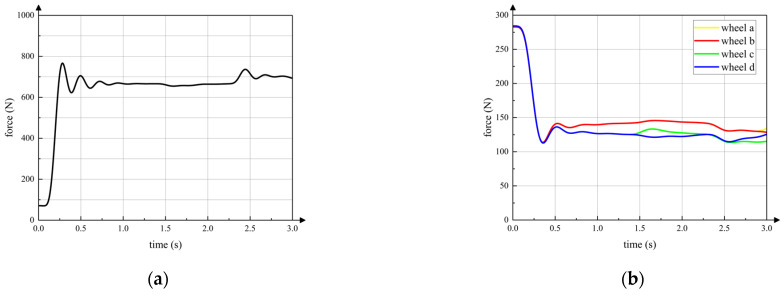
Vertical contact force between wheels and ground (unilateral passive wheels and active wheel on slope). (**a**) active wheel; (**b**) passive wheels.

Similarly, when unilateral passive wheels and the active wheel move on the gentle slope as shown in Figure 18, the vertical contact force of the active wheel and passive wheels are shown in Figure 19. The active wheel can still provide sufficient driving force for wheeled motion. To sum up, the control method of wheeled motion can be adopted on multiple terrains to ensure sufficient driving force and stable torso posture.

## 6. Conclusions

Addressing the problem that existing wheel-legged robot control methods are too complicated, this paper proposes a time-efficient control method of a wheel-legged robot based on velocity planning to solve both the wheeled trajectory control problem and gait planning and control problem through one control framework, which is named velocity planning control. TeCVP designs the control method for the stance phase and the swing phase of legged motion and wheeled motion, respectively, in accordance with the velocity planning and transformation of a rigid body. On that basis, leg sequences are planned for leg-to-wheel switching motion and wheel-to-leg switching motion. Through complexity analysis from two aspects of time complexity and instances of basic operations, it can be seen that TeCVP is more time-efficient than traditional VMC. In addition, simulations show that TeCVP can realize stable control of tripod gait motion, wheel-leg switching motion and wheeled motion. Combined with impedance control, TeCVP realizes soft interaction of the legged system with the environment to ensure robustness and protect hardware from damage through unexpected collisions. In addition, in accordance with the comparison of the running time between TeCVP and VMC, it can be obtained that the complexity of TeCVP is relatively low and the operating efficiency is significantly higher compared to VMC. 

As a whole, considering the simple structure of the wheel-legged robot and the advantages of TeCVP, such as lower complexity and better operating efficiency than VMC, the wheel-legged robot and control algorithm TeCVP have broad application prospects for future Mars exploration missions with respect to expanding the exploration region and prolonging the time of endurance. 

However, the simulations in this paper focus on relatively flat terrain and gentle slope. The terrain on Mars is much more complex and rugged. In order to make the proposed algorithm more suitable for application, the complex and unknown terrain of Mars will become the focus in future research and TeCVP and gait planning method will be improved to adapt to more rugged terrains, for example, steps, obstacles and deep pits. In addition, important work in further research is to develop a prototype of the hexapod wheel-legged robot and execute some experiments in order to prove the control method proposed in this paper.

## Figures and Tables

**Table 1:** Instances of Basic Operations of TeCVP.

Number of Equations	Instances of Basic Operations	
	Multiplication	Addition
(6)	0	4
(7)	4	0
(8)	2	3
(9)	0	2
(10)	4	2
(11)	4	2
Total	14	13

**Table 2:** Instances of Basic Operations During Main Process of VMC.

Stage of Operating	Instances of Basic Operations	
	Multiplication	Addition
Calculation of CoM desired force	8	12
LU decomposition	70	70
Calculation of joint torques	12	6
Total	90	88

**Table 3:** Coefficients of TeCVP.

	Coefficients	Value
stance phase	coefficient vector of sliding mode surface K	[1000;1000;1000;1000]
	damping vector of impedance control $K_{d}$	[10;10]
	stiffness vector of impedance control $K_{k}$	[200;200]
swing phase	damping vector of impedance control $K_{d}^{f}$	[100;100]
	stiffness vector of impedance control $K_{k}^{f}$	[3000;3000]
wheeled motion	coefficient vector of sliding mode surface $K^{s}$	[1000;1000]
	damping vector of impedance control $K_{d}^{s}$	[10]
	stiffness vector of impedance control $K_{k}^{s}$	[200]

**Table 4:** Coefficients of VMC.

Coefficients	Value
virtual stiffness vector of VMC $K_{vmc}^{k}$	[10,000;10,000;10,000;10,000]
virtual damping vector of VMC $K_{vmc}^{d}$	[100;100;100;100]

## Data Availability

Not applicable.
